# Optimization of Bromocriptine-Mesylate-Loaded Polycaprolactone Nanoparticles Coated with Chitosan for Nose-to-Brain Delivery: In Vitro and In Vivo Studies

**DOI:** 10.3390/polym15193890

**Published:** 2023-09-26

**Authors:** Mohamed M. Badran, Abdulrahman E. Alanazi, Mohamed Abbas Ibrahim, Doaa Hasan Alshora, Ehab Taha, Abdullah H. Alomrani

**Affiliations:** 1 Department of Pharmaceutics, College of Pharmacy, King Saud University, Riyadh 11451, Saudi Arabia; mbadran@ksu.edu.sa (M.M.B.); eelbadawi@ksu.edu.sa (E.T.); aomrani@ksu.edu.sa (A.H.A.); 2 Kayyali Chair for Pharmaceutical Industries, Department of Pharmaceutics, College of Pharmacy, King Saud University, Riyadh 11451, Saudi Arabia; aba@pharma.com.sa (A.E.A.); dalahora@ksu.edu.sa (D.H.A.)

**Keywords:** bromocriptine mesylate, chitosan coating, ex vivo permeation, optimization, poly ε-caprolactone nanoparticles

## Abstract

Bromocriptine mesylate (BM), primarily ergocryptine, is a dopamine agonist derived from ergot alkaloids. This study aimed to formulate chitosan (CS)-coated poly ε-caprolactone nanoparticles (PCL NPs) loaded with BM for direct targeting to the brain via the nasal route. PCL NPs were optimized using response surface methodology and a Box–Behnken factorial design. Independent formulation parameters for nanoparticle attributes, including PCL payload (A), D-α-tocopherol polyethylene glycol 1000 succinate (TPGS) concentration (B), and sonication time (C), were investigated. The dependent variables were nanoparticle size (Y1), zeta potential (Y2), entrapment efficiency (EE; Y3), and drug release rate (Y4). The optimal formulation for BM-PCL NPs was determined to be 50 mg PCL load, 0.0865% TPGS concentration, and 8 min sonication time, resulting in nanoparticles with a size of 296 ± 2.9 nm having a zeta potential of −16.2 ± 3.8 mV, an EE of 90.7 ± 1.9%, and a zero-order release rate of 2.6 ± 1.3%/min. The optimized BM-PCL NPs were then coated with CS at varying concentrations (0.25, 0.5, and 1%) to enhance their effect. The CS-PCL NPs exhibited different particle sizes and zeta potentials depending on the CS concentration used. The highest EE (88%) and drug load (DL; 5.5%) were observed for the optimized BM-CS-PCL NPs coated with 0.25% CS. The BM-CS-PCL NPs displayed a biphasic release pattern, with an initial rapid drug release lasting for 2 h, followed by a sustained release for up to 48 h. The 0.25% CS-coated BM-CS-PCL NPs showed a high level of permeation across the goat nasal mucosa, with reasonable mucoadhesive strength. These findings suggested that the optimized 0.25% CS-coated BM-CS-PCL NPs hold promise for successful nasal delivery, thereby improving the therapeutic efficacy of BM.

## 1. Introduction

Bromocriptine mesylate (BM) is a dopamine agonist derived from ergot alkaloids that is primarily used for managing the treatment of various disorders such as Parkinson’s disease, neuroleptic malignant syndrome, type 2 diabetes, acromegaly, and galactorrhea [1,2]. Although BM has therapeutic benefits, its oral administration is not preferred because of its low bioavailability. To overcome this limitation and achieve targeted drug delivery, intranasal (IN) administration of BM has gained popularity as a non-traditional route of drug delivery [3]. IN drug delivery offers advantages such as improved permeability and vascularity of the nasal mucosa, which facilitates systemic absorption of the drug [4]. Owing to its high blood flow rate and direct contact with the brain through the olfactory epithelium, the nasal cavity provides a potential pathway for drugs to reach the brain. However, the presence of metabolic, physical, and transporter-regulated barriers restricts the number of active substances whose concentrations can reach therapeutic levels in the central nervous system (CNS) [5]. Overcoming these challenges has led to the exploration of various approaches for enhancing the transport of drugs through biological barriers to reach the brain [6]. Nanoparticle carriers have emerged as successful strategies for delivering therapeutic molecules to the brain [7]. Polycaprolactone (PCL) nanoparticles have shown promising results as biodegradable carriers in drug delivery systems. They possess favorable characteristics such as non-toxicity, biocompatibility, biodegradability, and efficient drug transport capabilities [8]. Approval of PCL for therapeutic use by the U.S. Food and Drug Administration (FDA) further supports its safety and suitability for medical applications [9]. PCL, a polymer with a low melting point (60 °C) [10], has a wide range of potential applications and is highly recommended as a carrier for the delivery of various types of medications [11]. In numerous drug delivery applications, PCL NPs have been used successfully. Due to their high permeability, biocompatibility, and elimination from the body following bio-absorption, PCL NPs demonstrated excellent therapeutic [12]. The efficacy of controlled and targeted delivery of drugs was also shown by using PCL NPs. They have an intense ability to cross several physiological barriers [13]. Alex et al. [14] developed PCL NPs loaded with carboplatin with the intention of treating glioma through nasal delivery. Importantly, their study confirmed the safety and feasibility of utilizing PCL NPs through nasal application for targeting brain tissue. CS-coated PCL NPs loaded with dorzolamide were formulated to improve the drug’s ocular delivery [15]. They revealed the effective ocular drug delivery of dorzolamide with improved therapeutic activity. Rahat et al. [16] designed and proved the enhancement of the oral bioavailability of thymoquinone using CS-coated PCL NPs. Raval et al. [17] conducted a promising avenue for effectively treating lung cancer using CS-coated PCL NPs containing Silibinin. de Lima et al. [18] confirmed the potential utility of CS-decorated PCL NPs as a practical delivery carrier for 5-FU (5-fluorouracil) to enhance therapeutic management for head and neck squamous cell carcinoma. These features make PCL an excellent choice for formulating drug delivery systems that can effectively overcome biological barriers and deliver therapeutic agents to specific targets [10]. To enhance the nose-to-brain delivery of BM, the effects of surface coating of nanoparticles with poloxamer 188 and chitosan (CS) have been explored. Surface-coated nanoparticles show increased CNS penetration [19], and CS, a cationic polymer, is well-suited for nucleic acid delivery [20]. CS has been used as a carrier for delivering drugs from to the brain via the nasal route owing to its mucoadhesive properties, biocompatibility, antibacterial activity, biodegradability, and controlled drug release ability [21,22].

In this study, we aimed to formulate CS-coated PCL nanoparticles loaded with BM for use through the nasal route for direct drug targeting to the brain. The research objectives included designing and optimizing PCL nanoparticles loaded with BM, characterizing the nanoparticles, investigating the effect of the CS coating on particle properties and mucin interactions, studying ex vivo drug permeation, and analyzing the in vivo nose-to-brain delivery of BM in animal models. The results of this study provide insights into the development of an efficient and targeted drug delivery system for BM, potentially improving therapeutic efficacy while minimizing side effects.

## 2. Materials and Methods

### 2.1. Materials

BM was obtained from AMRI (Milan, Italy). Low-molecular-weight (LMW) chitosan (deacetylation degree of 91.5%), poly ε-caprolactone (PCL, Mw~14,000), in the form of homopolymer pellets, and D-α-tocopherol polyethylene glycol 1000 succinate (TPGS) were purchased from Sigma-Aldrich (Schnelldorf, Germany). Glacial acetic acid and methanol (high-performance liquid chromatography [HPLC] grade) were obtained from BDH (Leicestershire, UK). Dialysis bags (standard cellophane membrane; molecular weight cut-off equal to approximately 12,000) and methylene chloride were obtained from Sigma Chemical Co. (St. Louis, MO, USA). All the remaining chemicals were of analytical grade.

### 2.2. Experimental Design

The effects of the independent formulation parameters, including PCL payload (A), TPGS concentration (B), and sonication time (C), were studied using the Box–Behnken factorial design to predict the particle size (Y1), zeta potential (Y2), entrapment efficiency (EE; Y3), and drug release rate (Y4), as shown in Table 1. The PCL payload levels used were 20, 35, and 50 mg, respectively. Additionally, three concentrations of TPGS (0.03%, 0.165%, and 0.3% *w*/*v*) and three sonication times (2, 5, and 8 min) were investigated (Table 5 and Table 6). Design of experiment (DoE) was used to optimize different formulations; the software program Statgraphics Centurion (version 17.2.02) was adopted for this purpose, which suggested the 15 formulations that are listed in Table 1.

### 2.3. Preparation of Bromocriptine Mesylate-Loaded Nanoparticles

BM-NPs were prepared via solvent evaporation. Briefly, the formula weight of PCL was dissolved in 1 mL of the organic solvent (methylene chloride), and then BM was dissolved in the PCL solution. The TPGS aqueous phase was slowly added to the PCL solution, and the suspension was placed in an ice bath and dispersed using a probe sonicator (Thomas Scientific, Madison, WI, USA) for different time periods at a fixed voltage efficiency (60%). The obtained emulsion (o/w) was stirred by means of a magnetic stirrer for 3 h at room temperature and vacuum evaporated to remove any residues of the organic solvent. BM was separated from the aqueous phase via centrifugation at 13,000 rpm for 10 min, followed by double washing with distilled water [23,24]. The produced nanoparticles were lyophilized (Alpha 1-4 LD Plus; Martin Christ Gefriertrocknungsanlagen GmbH, Osterode am Harz, Germany). All formulations were prepared in triplicates.

### 2.4. Characterization of Bromocriptine Mesylate–Polycaprolactone Nanoparticles (BM-PCL NPs)

#### 2.4.1. Particle Size and Zeta Potential

The average particle sizes and zeta potential values of BM NPs bromocriptine mesylate–chitosan–polycaprolactone nanoparticles (BM-CS-PCL NPs) were measured after preparing dilutions of BM in double-distilled water by using the dynamic light scattering (DLS) technique using a Zetasizer analyzer (Nano-ZS90; Malvern Instruments, Worcestershire, UK). Particle size was determined using photon correlation spectroscopy and by analyzing electrophoretic mobility. The values represent the average values of three measurements, each with 10 replicates.

#### 2.4.2. Entrapment Efficiency (EE) and Drug Load (DL)

The EE% and DL% of BM in the prepared nanoparticles and BM-CS-PCL NPs were measured indirectly via centrifugation [25]. The NP suspensions were centrifuged at 30,000× *g* rpm and 4 °C for 30 min. The drug concentration in the supernatant was analyzed using HPLC, as described below. *EE*% and *DL*% were computed using the following equations, respectively [24].

(1) $$EE\;\left(\%\right)=\frac{Total\;drug-Free\;drug}{Total\;drug}\times 100$$

(2) $$DL\;\left(\%\right)=\frac{Total\;drug-Free\;drug}{Nanoparticle\;weight}\times 100$$

where *Total drug* is the amount of drug added, and free drug is the free amount of drug present in the supernatant solution.

#### 2.4.3. In Vitro Release of Bromocriptine Mesylate–Polycaprolactone Nanoparticles (BM-PCL NPs)

The in vitro drug release patterns from BM-loaded NPs and BM-CS-PCL NPs were obtained using a dialysis bag (molecular weight cut-off: approximately 12 kDa) that was soaked in distilled water for 12 h. A known weight of freeze-dried nanoparticle formulation equivalent to 1 mg of BM was redispersed in 1 mL of phosphate buffer (pH 7.4). The dispersion was then kept in a dialysis tube and immersed in 50 mL of the receptor fluid (phosphate buffer, pH 7.4). The tubes were shaken at 100 rpm and kept at 37 °C in a shaking water bath (Julabo Gmbh, Seelbach, Germany). Aliquots (2 mL) were withdrawn at predetermined intervals. The volume of the receptor solution was maintained at a constant by refilling with the same medium after each withdrawal to conserve the sink conditions. The amount of BM released was measured using HPLC after dilution. All experiments were performed in triplicates. 

#### 2.4.4. Analysis of Drug Release Kinetics

In vitro drug release data were fitted to different release kinetic models to determine the kinetics of drug release using different equations of zero- and first-order and Higuchi models. Consequently, a graph was plotted, which suggested that the diffusion mechanism followed either a Fickian or a non-Fickian mechanism. The Higuchi model describes drug release via a diffusion mechanism. “*n*” is calculated from linear regression of log (*Mt*/*M*) versus log *t*, where Mt/M is the fraction released by the drug at time t. If *n* = 0.45, Fickian diffusion is stipulated, whereas 0.45 < *n* < 0.89 suggests a non-Fickian diffusion mechanism. 

### 2.5. Chitosan Coating of the Optimized BM-PCL NPs

The optimized BM-PCL NPs were coated with CS through electrostatic interactions between the negatively charged uncoated BM-PCL NPs and positively charged CS coating solution, as described in previous studies [23,25]. In brief, a stock solution of CS was prepared at a concentration of 10 mg/mL by dissolving it in acetic acid (0.5%; *v*/*v*). Subsequently, a certain weight of the optimized BM-PCL NPs was mixed with a certain volume of the CS solution of various concentrations (0.25, 0.5, and 1%; *w*/*v*), resulting in the formation of CS-PCL NPs. The mixtures were stirred for 1 h at room temperature using a magnetic stirrer to facilitate coating. Subsequently, centrifugation was performed, and the resulting pellets were washed twice and redispersed in an equal volume of distilled water. The samples were centrifuged (5000 rpm for 5 min) to remove large particles or undissolved drugs from the formulations. Finally, the samples were freeze-dried at −60 °C for 3 days using a lyphilzer (Alpha 1–4 LD Plus, Martin Christ Gefriertrocknugs Anlagen GmbH, Osterode am Harz, Germany) [23,24]. To ensure reproducibility, all formulations were prepared in triplicates. The resulting nanoparticles were characterized based on their particle sizes, polydispersity indices (PDIs), zeta potentials, and EE%, as described in the previous sections.

### 2.6. Physicochemical Characterization of the Optimized BM-PCL NPs and BM-CS-PCL NPs

#### 2.6.1. Differential Scanning Calorimetry (DSC) 

The thermal behavior of the samples, including BM, PCL, CS, BM-PCL NPs, and BM-CS-PCL NPs, was analyzed using a Shimadzu DSC-60 instrument (Shimadzu Corporation, Tokyo, Japan). To conduct the analysis, samples weighing approximately 3–5 mg were placed in aluminum pans and sealed using a crimper to ensure proper containment. The sealed pans were subjected to a heating cycle starting at 25 °C, and the scanning temperature was regularly raised to 300 °C at a rate of 10 °C/min. Subsequently, the pans were cooled back down to 25 °C under a nitrogen atmosphere, which was adjusted to a flow rate of 40 mL/min. The thermal characteristics of the tested samples were investigated, which provided valuable insights into their thermal stabilities, phase transitions, and compatibilities.

#### 2.6.2. Powder X-ray Diffraction (PXRD) 

The crystallinity of BM, PCL, TPGS, CS, BM-PCL NPs, and BM-CS-PCL NPs was evaluated using an X-ray diffractometer (Rigaku Inc., Tokyo, Japan). The diffractometer utilized Cu Kα radiation for X-ray measurements. The PXRD patterns were obtained by scanning the samples over a 2θ range of 5–60°. This wide-angle range allowed the detection of diffraction peaks associated with the crystallographic structures present in the samples. 

Information on the crystallinity and crystalline phases was obtained by analyzing the positions and intensities of the diffraction peaks.

#### 2.6.3. Fourier-Transform Infrared Spectroscopy (FTIR) 

The FTIR spectra of the obtained formulations were recorded using an FTIR spectrophotometer (FT-IR Nicolet 380; Thermo Fisher Scientific, Madison, WI, USA). FTIR analysis was conducted to verify the possibility of chemical bonding between the drug and the polymer used. The FTIR spectra of the BM, PCL, TPGS, CS, BM-PCL NPs, and BM-CS-PCL NPs were recorded using an FTIR spectrophotometer. The compressed sample disc technique was employed by mixing a small quantity of the sample with potassium bromide using a hydraulic press. The obtained disc was scanned from 4000 to 600 cm^−1^.

#### 2.6.4. In Vitro Mucoadhesion

The ability of NPs to adhere to mucin, an essential component of the mucus layer, and demonstrate their mucoadhesive properties was evaluated via the in vitro mucoadhesion test. The mucoadhesive properties of the optimized BM and CS-coated BM were evaluated by determining their mucin-binding efficiencies, following the method described by Tzeyung et al. [26]. First, the bovine mucin powder was suspended in PBS (pH 7.4) at a concentration of 10 mg/100 mL. Known weights of the optimized BM-PCL NPs and CS-coated BM (50 mg) were added to 3 mL of mucin suspension. The dispersion was then vortexed for 1 min at room temperature to ensure interaction between the NPs and mucin. After an incubation period of 2 h, the zeta potential of the mucin suspension containing NPs was measured and compared with that of a pure mucin suspension. The change in the zeta potential value after the incubation period served as an indicator of the interaction between the NPs and mucin, reflecting their mucoadhesive properties [26,27]. These experiments were conducted in triplicates.

#### 2.6.5. Ex Vivo Permeation Study of BM-PCL NPs and BM-CS-PCL NPs

Freshly obtained nasal mucosa was carefully removed from the nasal cavity of goats procured from a local slaughterhouse. The tissue samples were positioned between the donor and receptor compartments of the Franz diffusion cell. The receptor compartment was filled with PBS (pH 7.4). To ensure appropriate oxygenation and agitation within the system, a mixture of 95% oxygen and 5% carbon dioxide gases was continuously bubbled through the receptor compartment [28]. The temperature was maintained at 37 °C throughout the experiments. After a pre-incubation period of 20 min, the optimized BM-PCL or BM-CS-PCL NPs were placed in the donor chamber of a diffusion cell. One mL of the sample was withdrawn from the receptor chamber at predetermined time intervals over 48 h. For each sampling, an equivalent volume of PBS medium was immediately replenished in the receptor chamber to maintain sink conditions [29]. The collected samples were analyzed using HPLC to determine the amount of BM that permeated the tissue.

Various permeation parameters, such as flux, permeation coefficient, and lag time, were determined to assess the permeation behavior of the drug through the nasal mucosa. These parameters are crucial for understanding the efficacy and potential applications of the developed formulations.

#### 2.6.6. Analysis of Surface Morphology

Lyophilized BM-PCL or BM-CS-PCL NPs were analyzed using scanning electron microscopy (SEM). The sample was sputter-coated with a thin layer of gold–palladium. Once the samples were appropriately coated, they were scanned using SEM. A voltage of 60 mV ensured optimal imaging conditions and provided detailed information on the surface morphology of the particles.

#### 2.6.7. HPLC Analysis of BM

The BM concentration was determined using a previously validated reverse-phase HPLC method with minor modifications [30]. The HPLC system (Waters^TM^ 600 controller; MA, USA) was monitored using “Empower (Water)” software. The mobile phase was prepared using sodium acetate and methanol (30:70; *v*/*v*), and the flow rate was set at 1.5 mL/min over a C_18_ column (Bondapak^TM^; 4.6 × 150 mm; particle size, 5 µm). The BM concentration in the withdrawn liquid samples was determined via UV detection at 300 nm with an injection volume of 10 µL at room temperature. For in vivo studies, BM in the plasma was extracted by mixing the samples with methanol using a vortexer and then drying at 37 °C. The dried samples were added to the mobile phase and filtered, and the amount of BM in the filtrate was measured using HPLC. 

### 2.7. Statistical Data Analysis

Analysis was conducted using Microsoft Excel (version 2010) and Origin software (version 8). The results are presented as mean ± standard error (*n* = 3), indicating that the experiments were performed in triplicates. One-way analysis of variance (ANOVA) was conducted to compare the results of experiments conducted under three or more conditions. This statistical test allows for the evaluation of significant differences among results obtained from multiple groups.

## 3. Results and Discussion

### 3.1. Effect of Formulation and Process Variables on PCL-Loaded BM Nanoparticle Size

The effects of individual variables (PCL payload [A], TPGS concentration [B], and sonication time) on the size of the prepared BM nanoparticles are shown in Table 2. 

**Figure 1:**
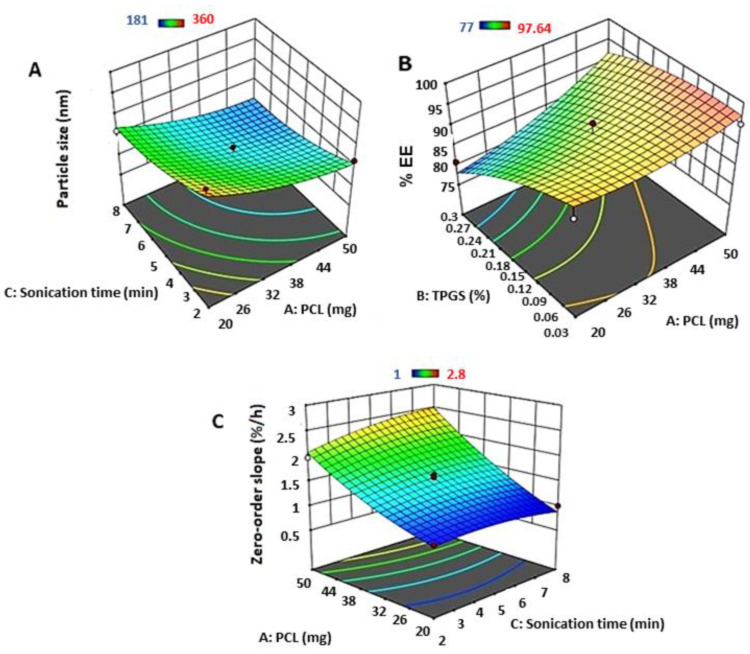
Response surface plots for the effect of independent parameters on (**A**) particle size, (**B**) zeta potential and (**C**) zeta potential of BM-loaded PCL nanoparticles.

*p*-values less than 0.05 represent the significant model terms, as shown in Table 2. The ANOVA results showed that PCL load and sonication time had a significant antagonistic effect on nanoparticle size (0.0057 and 0.0164, respectively). Other parameters, including TPGS concentration individually and interactive and quadratic effects, showed insignificant effects on nanoparticle size. The 3D response surface diagram for the impact of PCL load and sonication time is shown in Figure 1A. Increasing the PCL concentration and prolonging the sonication time resulted in a noticeable reduction in the BM nanoparticle size. The measured BM nanoparticle sizes are tabulated in Table 3. The BM-loaded PCL NP formulas showed particle sizes ranging from 215 to 360 nm, with a PDI range from 0.18 to 0.4.

The largest nanoparticle size (360 ± 5.4 nm) was recorded for nanoparticle formula F5, which was obtained using the lowest PCL load (20 mg), the highest TPGS load (0.3%), and the shortest sonication time (2 min). On the other hand, the smallest nanoparticle size (215 ± 3.02 nm) was observed for F2, which was formulated using the highest PCL load (35 mg), the lowest TPGS load (0.03%), and the longest sonication time (8 min). Rahayu et al. [31] showed that sonication resulted in a smaller agglomerated silica aerogel activated carbon (SA-AC) nanocomposite owing to the cavitation energy of ultrasound. Keum et al. [32] found that the smallest PLGA-loaded docetaxel nanoparticles were obtained upon increasing the sonication time. Thus, the sonication power likely decreased the nanoparticle size, probably because the sonication energy increased the energy released by emulsification and decreased the mean particle diameter. This could be considered a limiting factor, and even a small variation in sonication time alters the size of the NPs [33].

### 3.2. Effect of Formulation and Process Variables on PCL-Loaded BM Zeta Potential

The individual effects of A, B, and C on zeta potential are shown in Table 2. The ANOVA results showed that PCL load, TPGS concentration, and sonication time exerted insignificant impacts on nanoparticle zeta potential because the P values for these individual effects were higher than 0.05 (0.8305, 0.2060 and 0.2757, respectively). Other parameters, including interactive and quadratic effects, also showed insignificant effects on the nanoparticle zeta potential. The zeta potentials of the BM nanoparticles are tabulated in Table 3. The formulations exhibited zeta potentials ranging from 0.5 to 14 mV. The lowest zeta potential (0.5 mV) was recorded for nanoparticle formula F3, which was obtained using a medium PCL load (35 mg), the lowest TPGS load (0.03%), and the shortest sonication time (2 min). On the other hand, the highest zeta potential (−14 mV) was observed for F5, which was formulated using the lowest PCL load (20 mg), the highest TPGS load (0.165%), and the shortest sonication time (2 min). Pradhan et al. [34] found that prolonged sonication had negligible effects on the zeta potential of non-inert metal nanoparticles. However, Ref. [35] observed an increase in the negative zeta potential of nanoparticles with an increase in the concentration of PCL. Zhang et al. [36] found that NPs with a negative surface charge were more stable than those with a positive surface charge.

### 3.3. Effect of Formulation and Process Variables on PCL-Loaded BM Nanoparticle Entrapment Efficiency

Actual drug content values of all BM-loaded PCL nanoparticles loaded with BM were in the range of 77–97%.

The effects of individual variables (PCL load [A], TPGS concentration [B], and sonication time) on the entrapment efficiency of the prepared BM nanoparticles are shown in Table 2. The statistical data obtained from ANOVA indicated that TPGS exhibited an agonistic significant effect on the nanoparticle entrapment efficiency (*p* = 0.0079), whereas PCL exhibited a slightly significant antagonistic effect (*p* = 0.0440). Moreover, other parameters, including sonication time and interactive and quadratic effects, showed insignificant effects on entrapment efficiency. The 3D response surface plot for the effect of PCL load and TPGS concentration (Figure 1B) indicates that an increase in the PCL concentration led to a decrease in the EE % of BM inside the formulated PCL NPs. In contrast, an increase in the surfactant (TPGS) concentration increased the entrapment efficiency of BM nanoparticles inside the prepared nanoparticles.

The measured BM nanoparticle sizes are tabulated in Table 3. The formulations have EE ranging from 77.03 to 97.64%. The highest EE value (97.64 ± 3.79%) was recorded for nanoparticle formula F14, which was obtained using the highest TPGS load (0.3%) and the shortest sonication time (2 min) with the highest PCL load (50 mg). On the other hand, the lowest EE (77.03%) was observed for nanoparticle formula F1, which was formulated using a medium PCL load (35 mg), the lowest TPGS load (0.03%), and the shortest sonication time (2 min). These results revealed a prominent effect of TPGS concentration on the effect of PCL. These results are in agreement with the data obtained by Betala et al. [37], who reported that the entrapment efficiency of polymeric nanoparticles of the antihypertensive drug carvedilol increased upon increasing the polymer ratio. Al-Kordy et al. stated that TPGs have a significant positive effect on EE, which could be considered a limiting factor, and even a small variation in TPGs will alter the EE of NPs [33].

### 3.4. Effect of Formulation and Process Variables on In Vitro BM Release after 48 h from BM-Loaded PCL Nanoparticles

The in vitro BM release from its-loaded PCL nanoparticles was expressed as “zero-order release rate constant” or % BM released per min. The ANOVA statistical data for the effects of individual variables (PCL load [A], TPGS concentration [B], and sonication time [C]) on the zero-order release rate constant for the release of BM from the prepared nanoparticles are shown in Table 3. The ANOVA results showed that PCL loading had an extremely significant agonistic effect on the drug release rate from the nanoparticles (*p* = 0.0001). This might be explained by the antagonistic effect of PCL on the nanoparticle size, as mentioned previously in the section on particle size (Section 3.1). An increase in the PCL load resulted in a decrease in the nanoparticle size, which subsequently enhanced drug release. In addition, TPGS (quadratic effect; BB) showed a significant agonistic effect on drug release rate (*p* = 0.0438). Other parameters, including TPGS individually, sonication time, and interactive and quadratic effects, showed insignificant effects on drug release from nanoparticles. A 3D response surface plot of the effect of PCL loading and TPGS on the drug release rate from PCL-loaded BM nanoparticles is shown in Figure 1C. As mentioned previously, for the individual effects, PCL and the quadratic effect of TPGS enhanced drug release from these nanoparticle formulations. An increase in the PCL and TPGS concentrations resulted in a noticeable and significant increase in the drug release rate.

**Figure 2:**
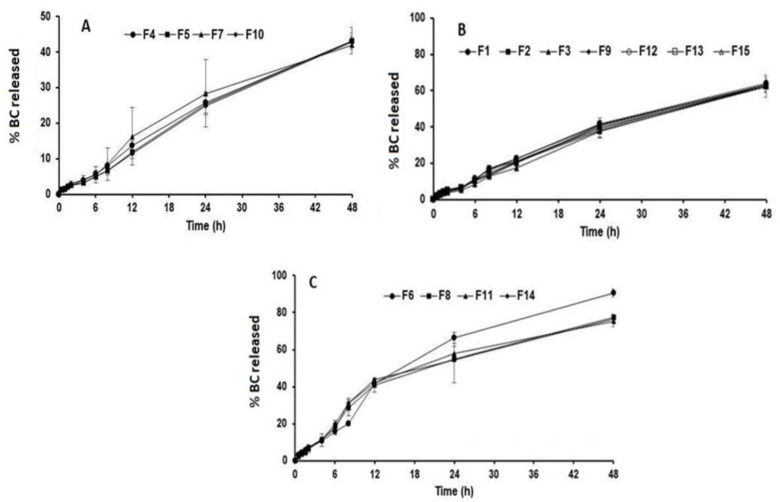
In vitro release of BM from PCL-loaded nanoparticle formulations containing low (**A**), medium (**B**) and high (**C**) PCL concentrations.

Drug release from different formulations of BM nanoparticles is shown in Figure 2. The formulations showed drug release ranging from 42 to 90.6%. Figure 2A shows the drug release patterns of the BM-loaded nanoparticles prepared using the lowest PCL concentrations (F4, F5, F7, and F10). The BM release patterns from the loaded nanoparticles prepared using formulations of medium PCL concentrations (F1, F2, F3, F9, F12, F13, and F15) are shown in Figure 2B. Figure 2C shows the BM release profiles of the loaded nanoparticles prepared using the highest PCL concentrations (F6, F8, F11, and F14). Initial burst release was not observed for NPs showing a monophasic pattern of drug release, and all NPs showed slow and sustained release behaviors. In addition, the drug release rate was the highest for nanoparticle formulations containing high levels of PCL, as clearly demonstrated by the results of the statistical analysis. The highest drug release (90.6%) was recorded for nanoparticle formula F6, which was obtained using the highest PCL load (50 mg), the highest TPGS load (0.3%), and a medium sonication time (5 min). In contrast, the slowest drug release rate (42%) was detected for F7, which was formulated using the lowest PCL load (20 mg), the lowest TPGS load (0.03%), and a medium sonication time (5 min). These data are in accordance with the data obtained from the particle size analysis, which indicated that PCL and TPGS exhibited antagonistic effects on nanoparticle size. Overall, an increase in the PCL and TPGS levels in the nanoparticles resulted in the formation of small nanoparticles, leading to enhanced drug release rates from such formulations. The release profile from the NPs is in line with the data reported by Ajiboye et al. [38].

Rezaei et al. [39] compared the dissolution rate of indomethacin nanoparticles with that of a physically micronized drug mixture containing polyvinyl pyrrolidone using a controlled pH-co-precipitation process. The nanosized version of the drug displayed a higher dissolution rate (45% in 30 min) compared to that of the mixed version of the microsized drug–PVP (10% in 30 min). They showed that the increased dissolution rate of indomethacin nanoparticles was related to a reduction in particle size, loss of crystalline form, and increased wettability owing to the presence of hydrophilic polymers. In another study, Liu et al. [40] altered the crystal environment and reduced the particle size to synthesize nanoparticles using celecoxib (CXB), because of which the CXB nanoparticles showed a markedly improved dissolution rate and oral bioavailability. Using nanoparticles, Kakran et al. [41] enhanced the dissolution rate of quercetin, a poorly water-soluble antioxidant. They used three methods to enhance the quercetin dissolution rate: solid dispersion with PVP and pluronic F127, complex formation with β-cyclodextrin, and fabrication of nanoparticles.

The data on in vitro release of BM from different PCL nanoparticle formulations were fitted using zero-order, first-order, and Higuchi diffusion models, as well as the Korsmeyer–Peppas equation, to obtain a model that could describe drug release from the loaded nanoparticles. The preference for the drug release mechanism was based on the correlation coefficient value. The data indicated a good fit to the zero-order kinetic release model for most of the 15 tested formulations (Table 4). Moreover, the “n” value derived from the Korsmeyer–Peppas equation was greater than 0.45, but all values were less than 1, indicating non-Fickian or anomalous drug release or the so-called coupled diffusion/polymer relaxation mechanism, which represents a combined effect of diffusion and erosion mechanisms for drug release [42]. 

As shown in Table 4, various kinetic models showed a linear relationship between the drug release rate and the drug concentration. The best linearity was observed using the zero-order equation, indicating that the drug showed a zero-order release mechanism from the tested NP formulations. The medicine dissolves in the medium as the core becomes hydrated until it reaches its saturation concentration (i.e., solubility). The core acts as a depleted drug reservoir. When a medication seeps through the membrane from the reservoir and subsequently diffuses across the membrane into the gastrointestinal fluid, it is released. The goal of all controlled-release drug delivery methods is zero-order release, in which a medication is released at a constant rate. In theory, this leads to the best plasma concentration management and has various advantages, including enhanced patient compliance and reduced drug delivery frequency. Although formulation scientists can utilize various approaches to obtain zero-order releases, most of these methods are sophisticated, expensive, and time-consuming, and it is difficult to manufacture nanoparticles using these methods. Furthermore, most formulation processes produce first-order release. Zero-order distribution kinetics refers to systems in which the rate of medication release remains constant over time. 

### 3.5. Optimization of Independent Parameters for Preparation of BM-Loaded PCL Nanoparticles

To optimize the BM-loaded PCL nanoparticle formulation before coating the nanoparticles with chitosan, the Statgraphics Centurion^®^ program was used for point prediction based on the desirability parameters. The desirability parameters were minimum particle size, maximum zeta potential, maximum EE, and maximum in vitro release rate (expressed using the zero-order release constant).

On the basis of the model developed using the statistical software and the desirability factor (95%), the following factors were suggested by the software for the preparation of optimal BM-loaded PCL nanoparticles: PCL (A) = 50 mg, TPGS (B) = 0.0865%, and sonication time (C) = 8 min. The predicted and observed values are listed in Table 5. Nanoparticles were then prepared using the optimized formula. Data regarding the nanoparticle size (Y1), zeta potential (Y2), EE (Y3), and zero-order release constant (Y4) are presented in Table 5. 

**Table 5:** Composition of the optimized BM-loaded PCL nanoparticle formula, the desirability of responses and their observed and predicted values.

Optimized Formula Composition	Response			
	Type	Desirability	Predicted	Observed
A: PCL (A) = 50 mg B: TPGS = 0.0865% C: Sonication time = 8 min	Y1: Particle size (nm)	Minimum	196.56	299.0 ± 2.9
	Y2: Zeta potential (mV)	Maximum	−14.64	−16.0 ± 1.07
	Y3: EE (%)	Maximum	92.97	90.7 ± 1.9
	Y4: Zero order release rate	Maximum	2.51	2.63 ± 1.3

The observed values were close to the predicted optimal values for the nanoparticle formula. The observed particle size was 296 ± 2.9 nm (the predicted particle size was 196.95 nm). The zeta potential value was observed to be −16.2 ± 3.84 mV, and the predicted value was −14.64 mV. Moreover, the EE value was observed to be 90.7 ± 1.9% (the predicted value was 92.97%), and the zero-order release constant was 2.63 ± 1.3%/h (the predicted value was 2.51). The optimized BM-loaded PCL nanoparticle formulation was then coated with chitosan and studied for ex vivo mucosal permeation.

### 3.6. Characterization of the Optimized BM-PCL NPs and BM-CS-PCL NPs 

#### 3.6.1. Particle size, Polydispersity Index, and Zeta Potential of Nanoparticles

Various strategies have been used to enhance the delivery of drugs from the nose to the brain using CS-coated nanocarrier systems. The size of these particles and their zeta potential are critical parameters that influence their ability to overcome the mucus barrier of the nasal membrane [43]. It has been reported that nanoparticles with a particle size range of 100 to 200 nm could facilitate efficient drug transport. However, large particles have demonstrated effective nose-to-brain delivery of various drugs [44,45]. In the current study, the optimized BM-PCL NP formulation exhibited a particle size of 299 ± 2.9 nm. In contrast, the particle size of BM-CS-PCL NPs increased significantly as the concentration of CS increased. Specifically, the sizes of BM-CS-PCL NPs were 331 ± 3.35, 425 ± 2.29, and 555 ± 5.19 nm at CS concentrations of 0.25, 0.5, and 1%, respectively (Table 6). These results clearly indicated that the particle size of the NP formula was controlled by the amount of CS utilized in the formulation, emphasizing the impact of the formulation variables on the final particle size [46].

**Table 6:** Properties of optimized bromocriptine mesylate–polycaprolactone nanoparticles (BM-PCL NPs) and bromocriptine mesylate-chitosan-polycaprolactone nanoparticles (BM-CS-PCL NPs).

Formula	Particle Size (nm)	Zeta Potential (mV)	PDI	EE%	DL%
BM-PCL NPs	299 ± 2.9	−16 ± 1.07	0.30 ± 0.35	90.7 ± 1.9	5.7 ± 0.8
BM-CS—0.25%	331 ± 3.35	21 ± 3.84	0.17 ± 0.26	87.9 ± 0.9	5.5 ± 0.5
BM-CS—0.5%	425 ± 2.29	25 ± 4.67	0.13 ± 0.01	85.2 ± 2.91	5.3 ± 0.4
BM-CS—1%	555 ± 5.19	30 ± 2.74	0.08 ± 0.018	82.7 ± 3.76	5.0 ± 0.7

PDI is an important parameter for the characterization of nanostructured platforms. Low PDI values, typically below 0.3, are preferred because they indicate a high degree of homogeneity in the nanoparticles. In the present study, the CS concentration had an insignificant effect on the PDI values of the obtained NPs, which ranged from 0.08 to 0.17 (Table 6). 

Zeta potential values are indicative of the stability of colloidal dispersions based on their magnitude [47]. The zeta potential can affect in vivo results, as the value is affected by interactions with the cell membrane. These effects on particle performance are relevant to nasal drug delivery. Positively charged nanoparticles tend to exhibit mucoadhesive properties that facilitate their adherence to the mucus layer. This mucoadhesive nature offers potential advantages, such as increased retention time in the nasal cavity, allowing for prolonged contact with the mucosa [48]. The zeta potential values obtained for the different formulations ranged from −16 to 30 mV (Table 6). The presence of CS in the formulations caused an alteration of the zeta potential from a negative to a positive sign. This might be due to the fact that a polyelectrolyte complex is formed between the NPs and the CS. Specifically, the zeta potential values for CS-coated BM-PCL NPs were 21 ± 3.84, 25 ± 4.67, and 30 ± 2.74 mV at CS concentrations of 0.25, 0.5, and 1%, respectively. This change in zeta potential can be attributed to the shielding effect of CS on the NP surface. Therefore, the amount of CS incorporated into the formulation plays a critical role in determining the physicochemical properties of the nanoparticles. CS may be arranged on the outermost surface of the NPs, with free amino groups producing positive zeta potential values. These positive zeta potential values indicate that CS effectively coated the surface of the BM-PCL NPs [49]. A possible mechanism for the CS coating process involves intermolecular hydrogen bonding between the hydroxyl groups of the PCL NPs and the amino groups of CS. It is important to note that this coating process can also increase the particle size because the CS molecules form a protective layer around the NPs [49].

#### 3.6.2. Entrapment Efficiency (EE) and Drug Loading (DL)

Comparison of BM-PCL NPs to BM-CS-PCL NPs prepared at different CS concentrations (0.25, 0.5, and 1%, respectively) revealed that the encapsulation efficiency (EE) of BM-PCL NPs was higher than that of BM-CS-PCL NPs. EE and DL of BM-CS-PCL NPs were 91% and 5.7%, respectively, whereas those of BM-CS-PCL NPs ranged between 83 and 88% and 5 and 5.5%, respectively (Table 6). This difference in EE can be attributed to the surface properties of the CS-coated nanocarriers [50]. Due to the hydrophobic nature of BM with a log *p* value of approximately 3.89, it is likely to be loaded into the hydrophobic core of the NPs. The process of coating NPs with CS solution significantly decreased the EE, particularly at 1% *w*/*v* CS concentration. Despite the reduction observed in EE following CS coating, the CS coating process can provide advantages such as limited drug burst release, reduced absorption of NPs by macrophages, and prolonged drug release from NP surfaces [51]. 

#### 3.6.3. Differential Scanning Calorimetry (DSC)

**Figure 3:**
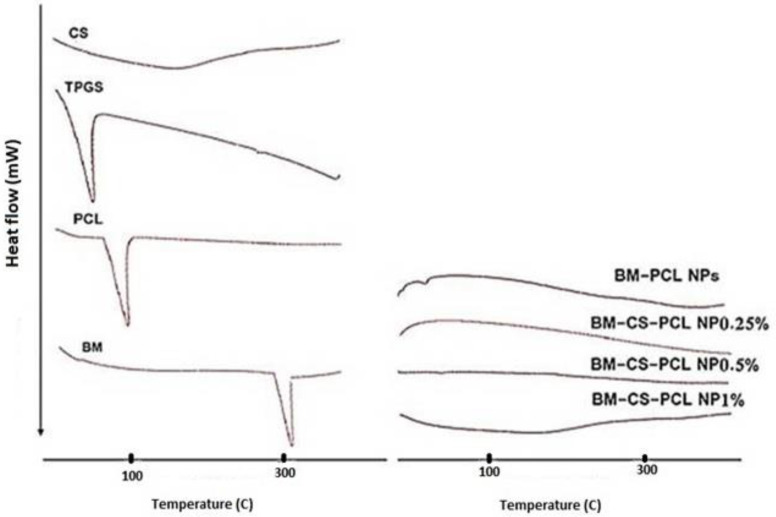
Differential scanning calorimetry (DSC) thermograms of optimized bromocriptine mesylate–polycaprolactone nanoparticles (BM-PCL NPs) and bromocriptine mesylate-chitosan-polycaprolactone nanoparticles (BM-CS-PCL NPs).

Figure 3 displays the DSC thermograms of BM, CS, PCL, TPGS, BM-PCL NPs, and BM-CS-PCL NPs (synthesized at 0.25% CS concentration). The DSC technique helps in detecting interactions between the drug and polymer within the NP structure. The DSC thermograms showed that the melting points of PCL, TPGS, and CS were 77.1, 61.2, and 91 °C, respectively. The thermogram of BM exhibited a distinct melting endothermic peak with an onset temperature of 209.10 °C, indicating its crystalline nature. However, these peaks were not observed in BM-PCL NPs and BM-CS-PCL NPs (synthesized at 0.25% CS concentration), suggesting the absence of crystalline BM outside the NPs. The drug’s crystallinity was significantly diminished or absent within the NPs. These results confirmed the successful loading of BM into the NPs, and it is likely that BM was uniformly dispersed within the PCL matrix.

#### 3.6.4. Powder X-ray Diffraction (PXRD)

**Figure 4:**
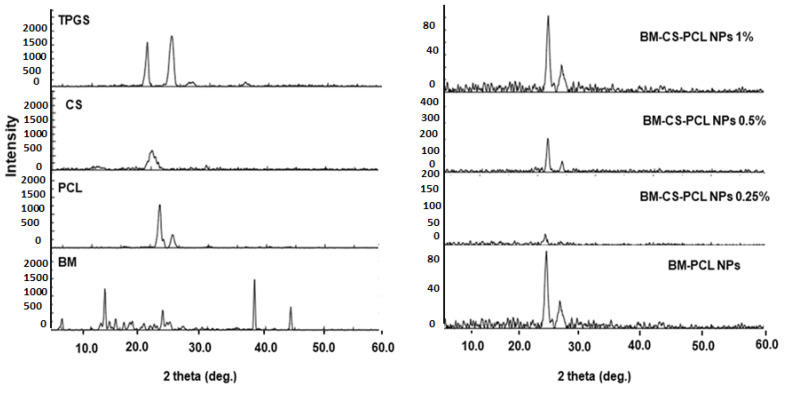
X-ray diffraction pattern of optimized BM-PCL NPs and BM-CS-PCL NPs.

As depicted in Figure 4, the X-ray pattern of BM exhibits three distinct peaks at 2-theta diffraction angles of 12°, 39°, and 47°, confirming the crystalline nature of the drug [52]. Furthermore, the X-ray diffractograms of PCL, TPGS, and CS exhibited characteristic peaks at 22°, 23°, and 19°, respectively, which is consistent with the findings of previous reports [26]. In the diffractogram of the optimized BM-PCL NPs, only two peaks with reduced intensities were observed at 2-theta diffraction angles of 22° and 23°. These peaks resembled those of PCL, whereas peaks corresponding to the drug were absent in this spectrum. This indicates successful encapsulation of BM within the PCL matrix and, possibly, amorphization of BM within the matrix [53]. The PXRD pattern of pure CS displayed very broad peaks at 2θ = 20°. However, the diffractogram of the optimized BM-CS-PCL NPs (0.25% CS coating) exhibited a weak peak at approximately 20°. Remarkably, the broad peak observed for CS at 2θ = 20° was weakened upon coating with CS. These findings suggest the good compatibility of CS, leading to a porous network formation within the NPs. The PXRD diffraction spectrum further denoted that the BM-CS-PCL NPs exhibited an amorphous form [54].

#### 3.6.5. Fourier-Transform Infrared Spectroscopy (FTIR) 

**Figure 5:**
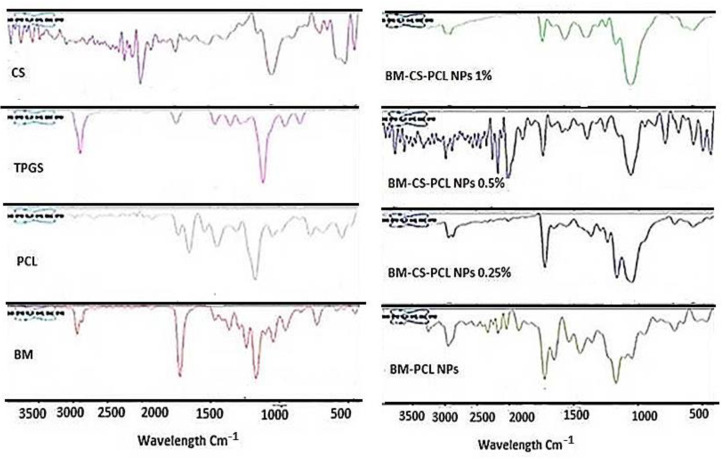
FTIR spectra of optimized BM-PCL NPs and BM-CS-PCL NPs.

Figure 5 shows the FTIR patterns of BM, PCL, TPGS, CS, BM-PCL NPs, and BM-CS-PCL NPs. The FTIR spectrum of BM exhibited characteristic peaks at 1649 cm^−1^ (C=O stretching), 1537 cm^−1^ (C=C–C aromatic ring stretching/N–H bending), 1170 cm^−1^ (amide III), 769 cm^−1^ (C–H), and 542 cm^−1^ (aliphatic C–I stretching), as reported by Md et al. (2012). The FTIR spectrum of PCL displayed intense peaks at 2939 cm^−1^ (O–H stretching), 1721 cm^−1^ (C=O stretching), 1167 cm^−1^ (amide III), and 581 cm^−1^ (C–I), indicating the characteristic vibrations of PCL. The FTIR spectrum of TPGS showed two sharp bands at 2880 cm^−1^ (C–H stretching) and 1111 cm^−1^ (C–F stretching). The FTIR spectrum of CS displayed prominent peaks at 2008 cm^−1^ and 1053 cm^−1^, verifying the existence of amide I and II vibrations [55]. The bands at approximately 1750 cm^−1^ (C=O stretching of amide I) indicated the presence of residual N-acetyl groups in CS. In the case of optimized BM-PCL NPs and BM-CS-PCL NPs, the characteristic bands of CS and TPGS were found in their positions unchanged. Moreover, the characteristic C=O stretching bands of BM and PCL were fund overlapped around 1700 cm^−1^ (Figure 4). In addition, the drug characteristic peaks that were observed in the spectrum of the pure drug were absent. This confirms the successful dispersion of BM within the nanoparticles, as reported by Tzeyung et al. [26].

#### 3.6.6. In Vitro Release of BM from CS-Coated NPs

**Figure 6:**
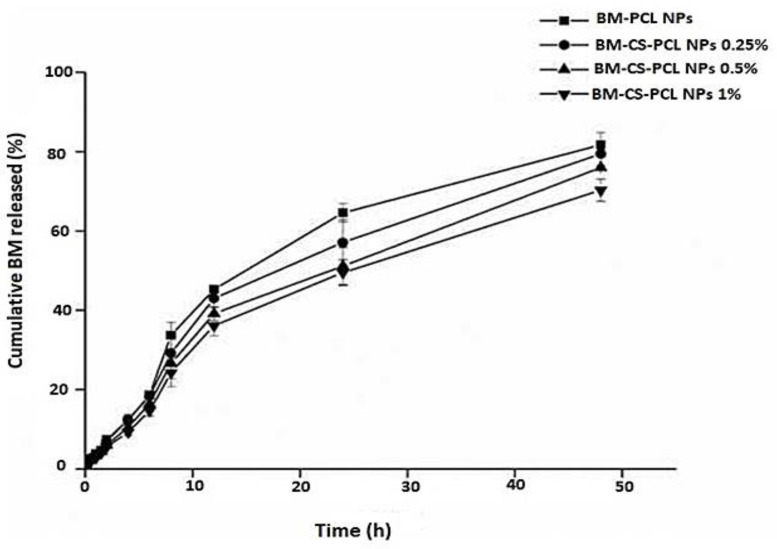
In vitro drug release pattern of optimized BM from BM-PCL NPs and BM-CS-PCL NPs.

The incorporation of CS surface modifications on polymeric NPs enhances the cellular uptake of a wide range of drugs. This modification was performed to promote efficient drug internalization by cells, thereby improving overall efficacy. The drug release profiles from different formulations of BM-PCL NPs and BM-CS-PCL NPs are displayed in Figure 6. BM-PCL and BM-CS-PCL NPs were able to control BM release because of the presence of two diffusional barriers: PCL polymer and positively charged CS [56,57].

The obtained formulations showed a sustained release effect lasting up to 48 h. BM-PCL NPs showed the highest level of drug release, whereas BM-CS-PCL NPs (synthesized at 1% CS concentration) showed the lowest. Thus, CS coating resulted in a significant modulation of in vitro drug release from BM-PCL NPs. Moreover, no significant change in the in vitro release of BM from BM-CS-PCL NPs (harboring 0.25 or 0.5% CS coating) was observed. PCL may reduce the initial penetration of water into the copolymer and suppress the release of BM from the NPs for 48 h [24]. 

The obtained results demonstrated that the NPs exhibited a sustained release pattern, with a gradual decrease in release rate over a period of 48 h, resulting in the release of 82% of the loaded BM. BM-CS-PCL NPs (harboring 1% CS coating) could significantly delay the release of BM, whose concentration reached 49.5 and 70.3% after 24 and 48 h, respectively. Therefore, the coating CS resulted in reduced drug release from the optimized NP formula.

#### 3.6.7. Drug Release Kinetics

Various kinetic models have been developed to describe the complete release of drugs from different dosage forms. These models established a relationship between the drug release rate and the drug concentration, as summarized in Table 7. Drug release from polymeric NPs is determined by a combination of processes, including drug desorption from the surface of NPs along with the erosion of the polymeric matrix. Therefore, drug transport through the NP matrix involves a combination of diffusion and erosion mechanisms. Table 7 presents a comparison of the in vitro drug release patterns predicted using various release models, such as zero-order, first-order, and Higuchi kinetics. For uncoated NPs, the first-order model exhibited a higher *R^2^* than that of the other models, indicating that the drug release profile of the uncoated NPs was predominantly determined by the diffusion process. The release data of the CS-PCL NPs were fitted to the widely recognized empirical equation of Korsmeyer and Peppas. The diffusion exponent, denoted as “*n*,” was determined to be within the range of 0.5 < *n* < 1, suggesting that the drug release from the system followed a non-Fickian release model. In this non-Fickian model, the drug release is influenced by both diffusion and swelling. Therefore, drug release from CS-coated NPs can be ascribed to a combination of diffusion and swelling mechanisms.

#### 3.6.8. Particle Surface Morphology

**Figure 7:**
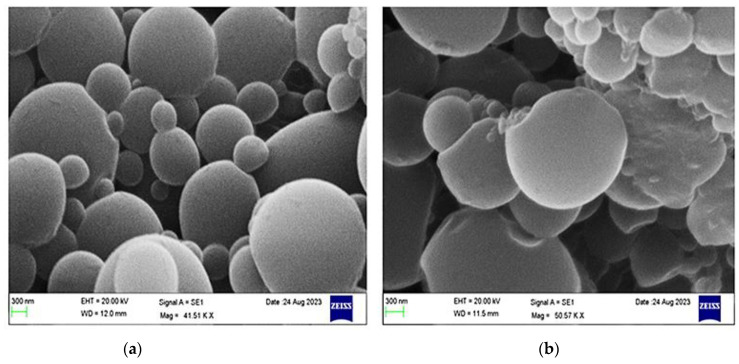
Scanning electron (SEM) micrographs of (**a**) bromocriptine mesylate–polycaprolactone nanoparticles (BM-PC NPs) and (**b**) bromocriptine mesylate–chitosan–polycaprolactone nanoparticles (BM-CS-PCL NPs; 0.25% chi-tosan [CS] coating). Bar = 300 nm.

The surface morphologies of BM-PCL NPs and BM-CS-PCL NPs (harboring 0.25% CS coating) were examined using SEM, as depicted in Figure 7. The SEM images revealed that the particles were smooth and spherical. However, the particles appeared slightly larger than those obtained by DLS, which can be attributed to the effect of lyophilization during sample preparation [58]. Owing to the interaction between CS and PCL NPs, a noticeable adhesion was observed as the amount of CS on the surface increased. This observation suggests that coating with CS led to an increase in particle diameter, as reported by Liu et al. [56].

#### 3.6.9. Analysis of Mucoadhesion

The mucoadhesive abilities of BM-PCL NPs and CS-coated BM-PCL NPs were indirectly assessed by evaluating their ability to adsorb mucin, a protein commonly secreted by mucosal epithelial tissues [59]. The surface charges of the obtained formulations were examined before incubating the formulations with a mixture of NPs and mucin. Following the incubation and removal of non-adsorbed mucin, the zeta potential was measured. The results of the in vitro bioadhesion study are presented in Table 8.

Mucoadhesive characteristics were influenced by the concentration of the bioadhesive polymer (CS). BM-PCL NPs and BM-CS-PCL NPs (0.25, 0.5, and 1% CS coating) exhibited zeta potential values of −29 ± 1.09, 7 ± 2.49, 8 ± 3.41, and 17 ± 1.67 mV, respectively, as shown in Table 8. The presence of mucin had a minimal effect on the negative surface charge of PCL NPs (*p* > 0.05). This can be attributed to the anionic charges and repulsive forces acting between BM-PCL NPs and mucin [59]. A noticeable change in the zeta potential of the CS-PCL NPs was observed when these NPs interacted with a small amount of mucin. These findings indicated that the bioadhesive behavior of NPs intensified as the concentration of CS increased [57]. This behavior confirmed the presence of electrostatic interactions between the negatively charged sialic acid residues of the mucin glycoprotein and the positively charged amino groups of CS [57]. Additionally, hydrogen bonding between the positively charged amino groups of CS and negatively charged mucin has been found to contribute to the mucoadhesive properties of the cationic polymer [58,60]. The positively charged CS-coated NPs promote greater adhesion and retention of the NPs [58]. 

Additionally, CS has been demonstrated to have the ability to temporarily open tight junctions and increase drug permeability across mucosal barriers [54]. This effect contributes to the enhanced bioadhesion and interaction of the CS-coated NPs with mucin owing to their positive zeta potential. The positive zeta potential facilitates the adhesion and retention of NPs on the negatively charged membranes and mucosa. Previous studies have emphasized the significance of the CS coating on BM-PCL NPs in effectively improving the mucosal residence time [61].

#### 3.6.10. BM Permeation across Excised Goat Nasal Mucosa

An ex vivo nasal mucosal permeation experiment was conducted to assess the efficacy of the formulations after nasal application. It has been reported that nasal absorption can be guided by several parameters, including nasal physiology, the physicochemical properties of the substance, and the type of formulation employed [62]. 

**Figure 8:**
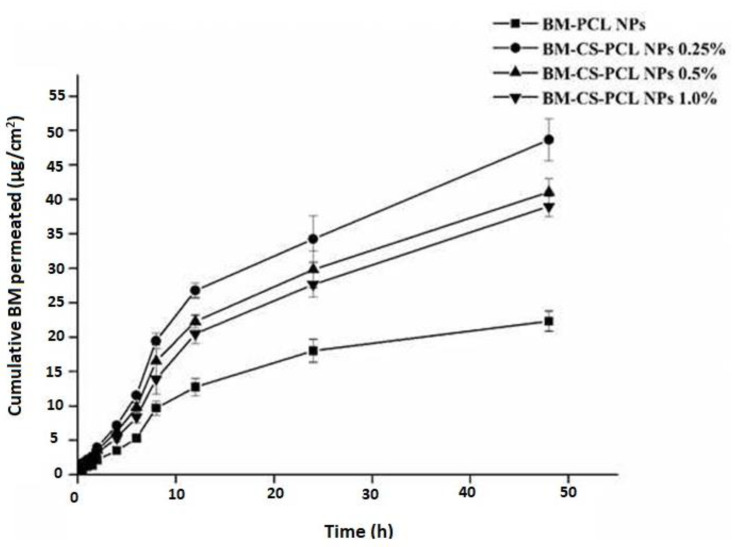
Ex vivo permeability patterns of bromocriptine mesylate–polycaprolactone nanoparticles (BM-PCL NPs) and bromocriptine mesylate–chitosan–polycaprolactone nanoparticles (BM-CS-PCL NPs).

Figure 8 represents the results of ex vivo permeation studies conducted using BM-PCL NPs and BM-CS-PCL NPs. The total amounts of the drug that permeated through the mucosa after 48 h from BM-PCL NPs and BM-CS-PCL NPs (0.25, 0.5, and 1% CS coating) were 22.290 ± 1.449, 48.655 ± 3.043, 41.049 ± 2.009, and 38.992 ± 1.469 µg/cm^2^, respectively. Among the formulations, the highest permeation was observed from BM-CS-PCL NPs (0.25% CS coating). These results indicated that BM-CS-PCL NPs showed a 2.2-fold higher nasal permeation compared to that of BM-PCL NPs. Overall, BM-CS-PCL NPs (0.25 % CS coating) showed enhanced nasal permeation of BM compared to that of other BM-PCL NP formulations. This could be attributed to the specific characteristics of the BM-CS-PCL NPs, such as their size, surface properties, and composition, which may facilitate efficient drug release and permeation across the nasal epithelium [62]. The enhanced permeability of BM-CS-PCL NPs (0.25% CS coating) might be explained based on the effect of CS on the surface modification of nanoparticles [63].

Furthermore, the enhanced permeability of BM-CS-PCL NPs (0.25% CS coating) could also be attributed to their high EE, which might improve the absorption and retention time of BM at the target site [63,64]. In addition, the mucoadhesive properties of CS facilitate its interaction with the mucosal surface and the disruption of tight junctions, resulting in improved paracellular transport [65]. These effects facilitate the transport of drugs across the nasal barrier and increase drug availability. These results show that the BM-CS-PCL NP formulation can enhance nasal drug delivery.

#### 3.6.11. Permeation Parameters

The intranasal permeation parameters of BM-PCL NPs and BM-CS-PCL NPs (0.25, 0.5, and 1% CS coating) were evaluated, and the results are presented in Table 9. According to the data shown in Table 9, BM-PCL NPs and BM-CS-PCL NPs (0.25, 0.5, and 1% CS coating) exhibited steady-state flux (*J*) values of 3.56 ± 0.18, 14.61 ± 2.23, 7.34 ± 0.63, and 6.52 ± 0.53 µg/cm^2^/h, respectively. The permeability coefficient values of BM for BM-PCL NPs and BM-CS-PCL NPs (0.25, 0.5, and 1% CS coating) were as follows: 35.538 ± 2.436, 147.58 ± 22.52, 74.66 ± 8.07, and 65.98 ± 6.98 cm/h, respectively. BM-CS-PCL NPs (0.25% CS coating) resulted in a 2.18-fold increase in BM permeability in comparison to the corresponding values of PCL NPs. The cumulative permeation parameters indicated that formulation composition played a critical role, leading to diverse intranasal permeation patterns. The positivity of the formulation increased the intranasal permeation of BM. This might be attributed to the effect of the CS coating, which facilitated the interaction of the NPs with the nasal mucosal membrane. 

## 4. Conclusions

The BM-CS-PCL NPs developed in this study demonstrated excellent outcomes in both in vitro and ex vivo analyses, indicating their potential for improved intranasal (IN) delivery. The nanoparticles were formulated and optimized using PCL, CS, and sonication using a Box–Behnken design, which was prepared based on 50 mg PCL, 0.0865% TPGS by using a sonication time of 8 min. This NP formula was coated with different CS solutions. The optimized BM-CS-PCL NPs (0.25% CS coating) exhibited a small particle size in the nano-range with high positive surface charge, an EE greater than 85%, and a spherical shape. These characteristics are favorable for efficient IN delivery and enhanced BM efficacy. The BM release profile from CS-PCL-NPs displayed a biphasic behavior, with an initial burst release observed during the first 2 h, followed by a sustained release lasting for up to 48 h. This controlled and sustained-release pattern is desirable for maintaining therapeutic drug levels over an extended period. The ex vivo study results demonstrated a significant enhancement in mucoadhesion and BM permeation across the goat nasal mucosa when BM-CS-PCL NPs (0.25% CS coating) were used compared to that observed for other formulations. This suggested improved drug permeation, potentially leading to enhanced therapeutic efficacy.

## Figures and Tables

**Table 1:** The independent parameters and dependent factors (responses) in Box–Behnken design for the formulation of BM nanoparticles by solvent evaporation method.

Independent Factors	Low	High	Unit
A: PCL	20	50	mg
B: TPGS	0.03	0.165	%
C: Sonication time	2	8	min
Dependent factors (responses)			
Particle size (Y1)			nm
Zeta Potential (Y2)			mV
Entrapment Efficiency (Y3)			%
In vitro release (Y4)			%

**Table 2:** ANOVA for the effects of independent factors on the properties of BM nanoparticles.

Response	Source	Sum of Squares	*p*-Value
Nanoparticle size (nm)	A: PCL	12,800.0	0.0057
	B: TPGs	325.125	0.4932
	C: Sonication time	7503.13	0.0164
	AA	780.776	0.3041
	AB	289.0	0.5171
	AC	196.0	0.5910
	BB	1507.85	0.1725
	BC	56.25	0.7710
	CC	1596.16	0.1625
Zeta potential (mV)	A: PCL	0.690313	0.8305
	B: TPGs	28.6525	0.2060
	C: Sonication time	20.3203	0.2757
	AA	1.95194	0.7201
	AB	3.2041	0.6477
	AC	49.2102	0.1153
	BB	7.86603	0.4809
	BC	12.6736	0.3784
	CC	7.26711	0.4973
EE (%)	A: PCL	105.786	0.0440
	B: TPGs	269.921	0.0079
	C: Sonication time	2.57645	0.6935
	AA	7.96379	0.4957
	AB	20.7435	0.2892
	AC	61.7796	0.0962
	BB	19.5557	0.3018
	BC	0.0225	0.9704
	CC	20.8905	0.2876
Zero order release constant (%/h)	A: PCL	3.76751	0.0001
	B: TPGs	0.0120125	0.4480
	C: Sonication time	0.03645	0.2112
	AA	0.0732333	0.0979
	AB	0.0289	0.2579
	AC	0.055225	0.1379
	BB	0.12751	0.0438
	BC	0.005625	0.5977
	CC	0.0546564	0.1396

**Table 3:** Particle size and zeta potential values of BM-loaded PCL nanoparticle.

#	PCL (mg)	TPGS (%)	Sonication Time (min)	Particle Size (nm)	Zeta Potential (mV)	BM Content (%)	EE (%)
**F1**	35	0.3	2	261 ± 7.07	−10.7 ± 1.03	97.01	77.03 ± 3.06
**F2**	35	0.03	8	215 ± 3.02	−9.78 ± 2.3	95.71	95.79 ± 5.9
**F3**	35	0.03	2	245 ± 2.89	0.5 ± 4.65	93.60	93.79 ± 1.3
**F4**	20	0.3	5	241 ± 3.54	−3.62 ± 5.4	84.21	91.0 ± 2.9
**F5**	20	0.165	2	360 ± 5.4	−14 ± 1.17	82.0	82.72 ± 1.9
**F6**	50	0.3	5	256 ± 3.64	−7.3 ± 3.0	93.51	93.04 ± 6.8
**F7**	20	0.03	5	292 ± 3.12	−1.97 ± 1.76	92.61	92.14 ± 4.8
**F8**	50	0.165	8	232 ± 3.35	−11.3 ± 3.89	92.61	84.9 ± 3.1
**F9**	35	0.165	5	217 ± 2.9	−11.5 ± 2.47	93.74	93.1 ± 7.6
**F10**	20	0.165	8	261 ±3.89	−9.6 ± 3.69	95.07	90.74 ± 1.09
**F11**	50	0.03	5	230 ± 2.26	−7.9 ± 1.43	90.08	95.08 ± 2.69
**F12**	35	0.3	8	216 ± 5.23	−12 ± 1.08	87.21	82.3 ± 0.9
**F13**	35	0.165	5	258 ± 2.88	−5.16 ± 2.74	77.10	87.39 ± 1.11
**F14**	50	0.165	2	263 ± 2.12	−1.77 ± 3.19	79.80	97.64 ± 3.79
**F15**	35	0.165	5	226 ± 1.54	−8.89 ± 2.89	81.02	92.96 ± 2.69

**Table 4:** Model fitting results of BM release from its-loaded.

Formulation	Zero Order		First Order		Higuchi		Korsmeyer-Peppas	
	r	Slope	r	Slope	r	Slope	n	r
**F1**	0.996	1.714	−0.997	−0.010	0.960	8.416	0.757	0.984
**F2**	0.996	1.662	−0.997	−0.009	0.961	8.177	0.812	0.989
**F3**	0.997	1.530	−0.992	−0.008	0.941	7.358	0.809	0.986
**F4**	0.998	1.070	−0.997	−0.005	0.946	5.173	0.842	0.989
**F5**	0.994	1.021	−0.990	−0.005	0.926	4.850	0.768	0.973
**F6**	0.991	2.803	−0.989	−0.020	0.949	13.683	0.813	0.986
**F7**	0.993	1.187	−0.992	−0.006	0.939	5.725	0.824	0.982
**F8**	0.971	2.425	−0.987	−0.015	0.973	12.392	0.860	0.990
**F9**	0.998	1.584	−0.998	−0.009	0.961	7.783	0.731	0.989
**F10**	0.994	0.997	−0.990	−0.005	0.925	4.734	0.751	0.973
**F11**	0.970	2.577	−0.987	−0.017	0.969	13.120	0.901	0.990
**F12**	0.988	1.694	−0.999	−0.009	0.979	9.505	0.746	0.985
**F13**	0.998	1.541	−0.998	−0.008	0.960	7.562	0.735	0.991
**F14**	0.927	1.969	−0.945	−0.011	0.969	10.504	0.845	0.987
**F15**	0.998	1.627	−0.994	−0.009	0.952	7.911	0.721	0.989

**Table 7:** In vitro release kinetics of BM from BM-PCL NPs, and BM-CS-PCL NPs.

Formulation	Zero-Order		First Order		Higuchi		Korsmeyer-Peppas	
	R^2^	Slope	R^2^	Slope	R^2^	Slope	n	R^2^
BM-PCL NPs	0.975	2.631	0.993	0.017	0.968	13.346	0.852	0.991
BM-CS-PCLNPs0.25%	0.970	2.331	0.987	0.014	0.971	11.918	0.826	0.990
BM-CS-PCL NPs0.5%	0.968	2.097	0.983	0.012	0.969	10.732	0.835	0.991
BM-CS-PCL NPs1%	0.975	2.028	0.987	0.011	0.968	10.288	0.871	0.994

**Table 8:** Mucin mucoadhesion of BM-PCL NPs and CS coated BM-PCL NPs.

Formulae	Mucoadhesion with Mucin Interaction
BM-PCL NPs	−29 ± 1.09
BM-CS-PCLNPs 0.25%	7 ± 2.49
BM-CS-PCL NPs 0.5%	8 ± 3.41
BM-CS-PCL NPs 1%	17 ± 1.67

**Table 9:** Ex vivo permeation parameters of bromocriptine mesylate–polycaprolactone nanoparticles (BM-PCL NPs) and bromocriptine mesylate–chitosan–polycaprolactone nanoparticles (BM-CS-PCL NPs).

Formulations	BM Permeated	*J* (µg/cm^2^/h)	*Kp* × 10^−4^ (cm h^−1^)	ER
BM-PCL NPs	22.29 ± 1.45	3.56 ± 0.18	35.54 ± 2.44	-
BM-CS-PCL NPs 0.25%	48.65 ± 3.04	14.61 ± 2.23	147.59 ± 22.52	2.18
BM-CS-PCL NPs 0.5%	41.05 ± 2.01	7.34 ± 0.63	74.66 ± 8.07	1.84
BM-CS-PCL NPs 1%	38.99 ± 1.47	6.52 ± 0.53	65.98 ± 6.98	1.75

ER: Enhancement ratio; *J*: Permeability flux; *Kp*: Permeability coefficient.

## Data Availability

Data are contained within the article.
